# Neurite regrowth stimulation by a red-light spot focused on the neuronal cell soma following blue light-induced retraction

**DOI:** 10.1038/s41598-019-54687-w

**Published:** 2019-12-03

**Authors:** Yu-Chiu Kao, Yu-Cing Liao, Pei-Lin Cheng, Chau-Hwang Lee

**Affiliations:** 1 Research Center for Applied Sciences, Academia Sinica, Taipei, 11529 Taiwan; 2 Institute of Biophotonics, National Yang-Ming University, Taipei, 11221 Taiwan; 3 Institute of Molecular Biology, Academia Sinica, Taipei, 11529 Taiwan

**Keywords:** Neurology, Biophysical methods

## Abstract

The interaction of light with biological tissues has been considered for various therapeutic applications. Light-induced neurite growth has the potential to be a clinically useful technique for neuron repair. However, most previous studies used either a large illumination area to accelerate overall neurite growth or employed a light spot to guide a growing neurite. It is not clear if optical stimulation can induce the regrowth of a retracted neurite. In the present work, we used blue light (wavelength: 473 nm) to cause neurite retraction, and we proved that using a red-light (wavelength: 650 nm) spot to illuminate the soma near the junction of the retracted neurite could induce neurite regrowth. As a comparison, we found that green light (wavelength 550 nm) had a 62% probability of inducing neurite regrowth, while red light had a 75% probability of inducing neurite regrowth at the same power level. Furthermore, the neurite regrowth length induced by red light was increased by the pre-treatment with inhibitors of myosin functions. We also observed actin propagation from the soma to the tip of the re-growing neurite following red-light stimulation of the soma. The red light-induced extension and regrowth were abrogated in the calcium-free medium. These results suggest that illumination with a red-light spot on the soma may trigger the regrowth of a neurite after the retraction caused by blue-light illumination.

## Introduction

Controlling neurite growth is an essential technique in neuroscience, developmental biology, biophysics, and biomedicine; it is particularly important for the formation of neural circuits *in vitro*, as well as nerve regeneration *in vivo*. Many chemical cues that mediate axon and neurite growth have been identified, although their interplay is rather complex^1–4^. These biochemical signals eventually affect the actin cytoskeleton, which advances the leading edge of a growth cone through the polymerization of actin filaments and interactions with specific motor molecules^5–7^. Growing neurites are attracted to or retracted from various external stimuli and guidance factors. While chemical guidance factors have been studied intensively^8^, biochemical–mechanical coupling in growth cone regulation has also been noticed to play important roles in neurite growth^9^. Mechanical stress has also been found to induce neurite retraction with calcium-dependent signalling^10^. Chemical and physical factors that influence the polymerization and disassembly of microtubules, actin filaments, and neurofilaments may significantly alter the growth and stabilization of axons^11^. Hence, one may hypothesize that combining chemical and physical stimulations to control neurite growth should be a useful technique for the repair of neural networks. However, most chemical and physical stimulations cannot achieve subcellular localization accuracy and are irreversible.

Among various physical factors that could influence neurite growth, light illumination is of interest because it can be introduced to the damaged neural tissues in a flexible and less intrusive manner. A number of optical stimulation techniques have been proposed for attracting or guiding growing neurites with high spatial accuracy and effectiveness^12–20^. On the other hand, blue light (wavelength: 400–500 nm, intensity: 0.1–10 mW/mm^2^) induces the production of reactive oxygen species in cells and causes detrimental effects^21,22^ or suppresses cell proliferation^23,24^. Optical treatments have the capability of subcellular localized stimulation, which is difficult in chemical and electrical treatments. For example, controlling the extension and retraction of a specific lamellipodium on a single cell with focused laser light spots has been demonstrated^25^. These previous works suggest that optical stimulation holds the potential to be a versatile control technique in neurite damage and regrowth.

In the present work, we investigated the effects of blue and red light on neurite retraction and regrowth. We used a focal spot of 473 nm blue light and 650 nm red light to illuminate the neurite tips and soma of mouse neuroblastoma cells (N2a), respectively. The blue light caused neurite retraction which mimicked the pathological neurite degradation seen in neuron injury or neurodegenerative diseases. We found that the red-light spot on the soma induced the regrowth of retracted neurites. Furthermore, neurite extension and regrowth lengths were increased by the pre-treatment with a myosin II inhibitor, blebbistatin (BBI). Interestingly, we observed actin propagation toward the tip of growing neurites when we used the red-light spot to illuminate the soma. The optically induced neurite extension and regrowth did not occur in calcium-free medium. In addition to the results obtained with N2a cells, red light-induced neurite regrowth and actin propagation in neurites were also observed in primary rat hippocampal neurons.

## Results

### Blue light causes neurite retraction

**Figure 1:**
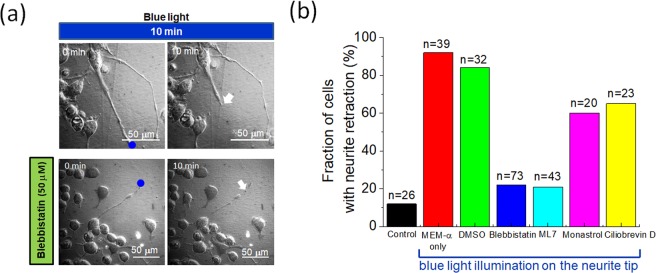
(**a**) Neurite retraction of N2a cells caused by a 473 nm blue-light spot. The blue dots are the locations of the 473 nm light spots on the neurites. The arrows indicate the tips of the illuminated neurites after 10 min. For the cell at the top, the blue-light spot at the neurite tip induced a 56 μm retraction in 10 min. For the cell at the bottom, with the pre-treatment of 50 μM blebbistatin (BBI), the neurite retraction in 10 min was 15 μm. (**b**) Probability of N2a cells exhibiting neurite retraction caused by blue light for pre-treatments with BBI, ML7, monastrol, and ciliobrevin D. The pre-treatments with BBI and ML7 reduced the fraction of cells with neurite retraction most obviously. The ‘control’ group refers to those cells with no optical or chemical stimulation. A retraction event was only recognized for neurites which retracted more than 5.0 μm after 10 min of blue-light illumination plus a 30-min duration without illumination. The original data for generating (**b**) are provided in the Supplementary Information.

We first used a 473 nm blue-light spot at the tip of a neurite to cause neurite retraction in N2a cells. As shown in Fig. 1(a), the blue-light spot at the tip of a neurite induced a 56 μm retraction in 10 min. In contrast, for the cells without blue-light stimulation, we barely observed identifiable retraction in 10 min. We therefore suspected that the blue light-induced neurite retraction could be driven by motor proteins. Using inhibitors for myosin II (BBI), myosin light chain kinase (ML7), kinesin-5 (monastrol), and dynein (ciliobrevin D) to pre-treat the N2a cells, we found that BBI and ML7 were able to reduce the probability of neurite retraction from 92% to 22% and 21%, respectively. In comparison, monastrol and ciliobrevin D decreased the neurite retraction probability to 60.5% and 65%, respectively Fig. 1(b). In the medium containing only the solvent of these inhibitors (DMSO, 1% v/v), the neurite retraction probability was 84%. From these results, we presumed that myosin in neurites might be the major driving molecule in blue light-induced retraction. In the following experiments, we included BBI pre-treatment to see if the inhibition of myosin II activity could help optically induced neurite regrowth.

### Effects of different wavelengths and power levels on neurite regrowth

**Figure 2:**
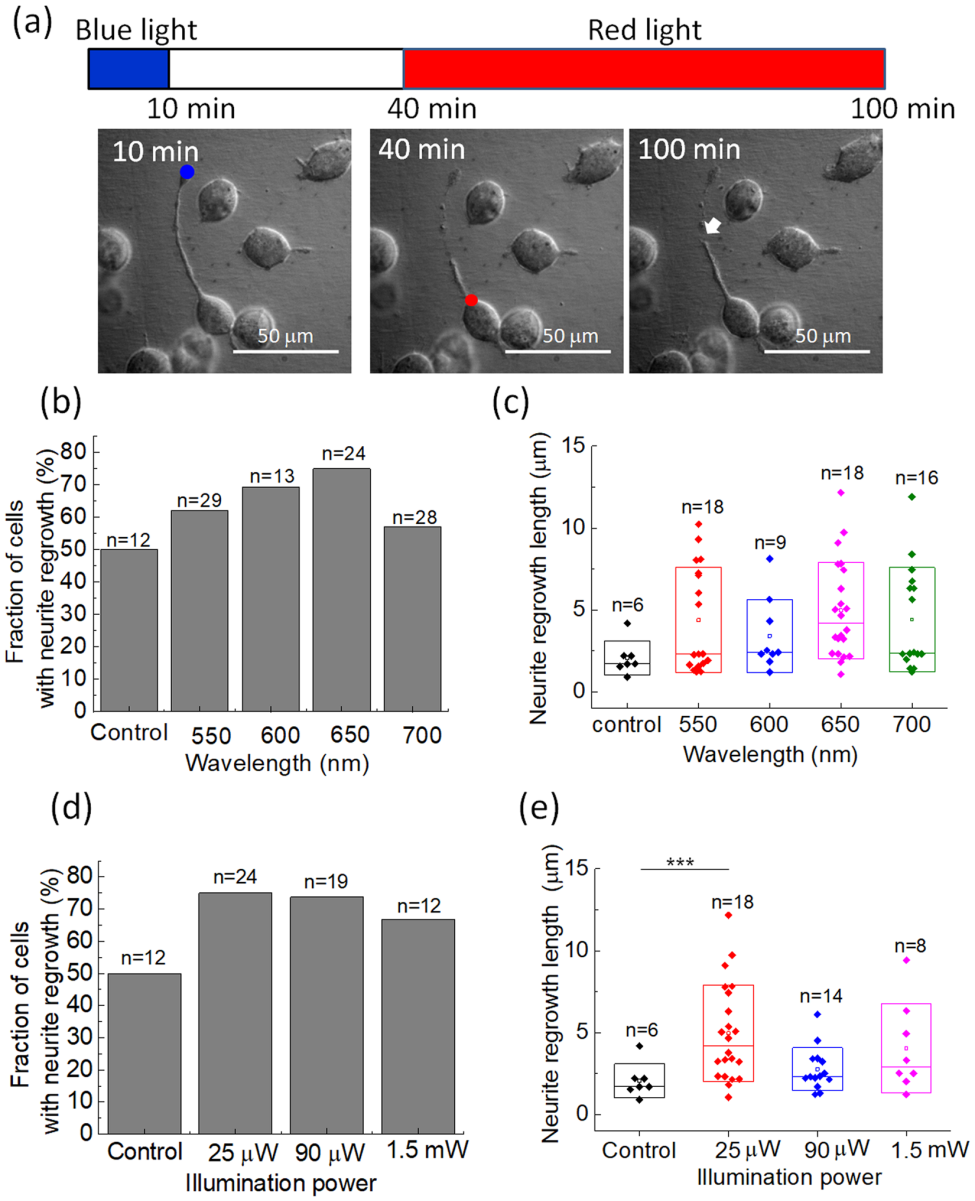
(**a**) Temporal sequence of the red light-induced neurite regrowth experiment of N2a cells. We first illuminated the tip of a neurite with blue light for 10 min, and then left the cells unilluminated for 30 min. We used a focused laser light spot to illuminate the soma close to the junction of the retracted neurite, as marked by the red dot in the middle photo. A regrowth event was only recognized for neurites with growth lengths of more than 1.0 μm after 60 min of illumination. (**b**) Probability of N2a cells exhibiting neurite regrowth under illumination of various wavelengths on soma. The illumination power on the cells for all wavelengths was 25 μW. (**c**) Neurite regrowth lengths in 1 h under various wavelengths of illumination. (**d**) Probability of N2a cells exhibiting neurite regrowth under various power levels of the 650 nm light. (**e**) Neurite regrowth lengths in 1 h under various power levels of the 650 nm light. ‘Control’ represents those cells subjected to 10 min of blue-light illumination only. ***Significant at α < 0.005 (post-hoc Scheffe’s test). The original data for generating (**b,d**) are provided in the Supplementary Information.

We explored the possibility of light-induced neurite regrowth in N2a cells after 473 nm blue light-induced retraction. The blue light was irradiated at the tip of a neurite for 10 min, and then the red-light spot was illuminated on the soma at the neurite junction after 30 min of rest time, as shown in Fig. 2(a). We compared the effects on neurite regrowth of various wavelengths [Fig. 2(b,c)] and power levels of 650 nm light [Fig. 2(d,e)]. Without any optical stimulation, about 50% of the retracted neurites exhibited regrowth. Using a focused light spot to illuminate the soma close to the junction of the retracted neurite, we found that 650 nm light had the highest probability (75%) of inducing neurite regrowth at a power of 25 μW. In comparison, 550 nm and 700 nm light had a 62% and a 57% probability of inducing the regrowth, respectively. However, there was no significant differences in neurite regrowth lengths after 1 h of illumination among the wavelengths tested Fig. 2(c). We also tested the effects of different power levels of 650 nm light and found that 25 μW illumination on the soma produced the most significant results for neurite regrowth [Fig. 2(d,e)]. Therefore, we used this power setting for the following experiments. However, because we did not search for the optimal power level for each wavelength, it is possible that other wavelengths might also be as effective at different power levels.

### Effects of BBI pre-treatment on red light-induced neurite regrowth

**Figure 3:**
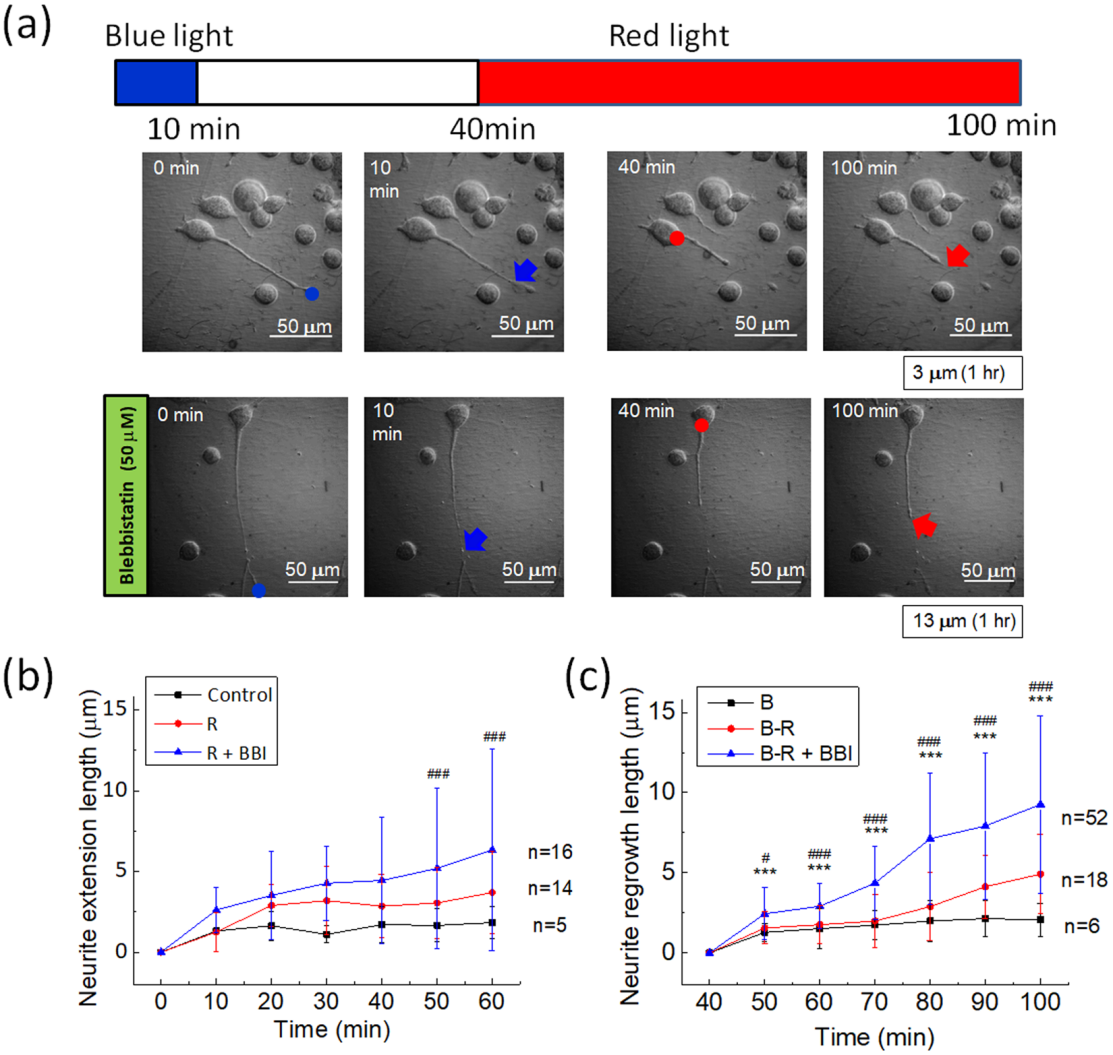
Effects of red light and BBI pre-treatment on the extension and regrowth of neurites of N2a cells after blue light-induced retraction. (**a**) Neurite retraction caused by a 473 nm blue-light spot at the tip, and the regrowth caused by a 650 nm red-light spot on the soma. The neurite regrowth length of this cell was 3 μm for 60 min of red-light illumination. In the other experiment with 50 μM BBI pre-treatment, the neurite regrowth length was 13 μm in 60 min. (**b**) Temporal variations of average neurite extension under red-light illumination (R) with and without BBI. The red-light illumination was maintained during the whole measurement. An extension event was only recognized for neurites which grew more than 1.0 μm after 60 min of illumination. Error bar, standard deviation. ^###^Significant at α < 0.005 (post-hoc Scheffe’s test) between the control group and the group under red-light stimulation with BBI pre-treatment (R + BBI). (**c**) Temporal variations of average neurite regrowth under red-light illumination (R) with and without BBI after blue-light illumination (**b**). The optical stimulation was applied as indicated by the timing bar in panel (a), and here shows the results after the red light being turned on. Error bar, standard deviation. ^#^Significant at α < 0.05 and ^###^Significant at α < 0.005 (post-hoc Scheffe’s test) between the group under blue-light stimulation only (**b**) and the group under blue- and red-light stimulation with BBI pre-treatment (B−R + BBI). ***Significant at α < 0.005 (post-hoc Scheffe’s test) between the two groups under blue- and red-light stimulation without (B–R) and with BBI pre-treatment (B–R + BBI).

From the results in Figs. 1 and 2, we postulated that myosin II could be the major source of the neurite retraction force and that red-light stimulation on the soma is beneficial for neurite regrowth. We compared the neurite regrowth induced by red light without and with BBI pre-treatment in N2a cells, as shown in Fig. 3. With the pre-treatment of 50 μM BBI, the retracted neurites exhibited a longer regrowth in 60 min compared to neurites with red-light illumination only, as shown in Fig. 3(a). We also show the temporal variations of average extension Fig. 3(b) and regrowth length Fig. 3(c) of neurites without and with blue-light illumination. For the data in Fig. 3(b), the N2a cells were under red-light stimulation from the beginning. In Fig. 3(c), the experiments were conducted according to the timing bar in Fig. 3(a) but we show only the regrowth lengths during the red-light illumination period. The results indicate that the BBI pre-treatment increases the lengths of red light-induced neurite extension as well as regrowth after retraction caused by blue light.

**Figure 4:**
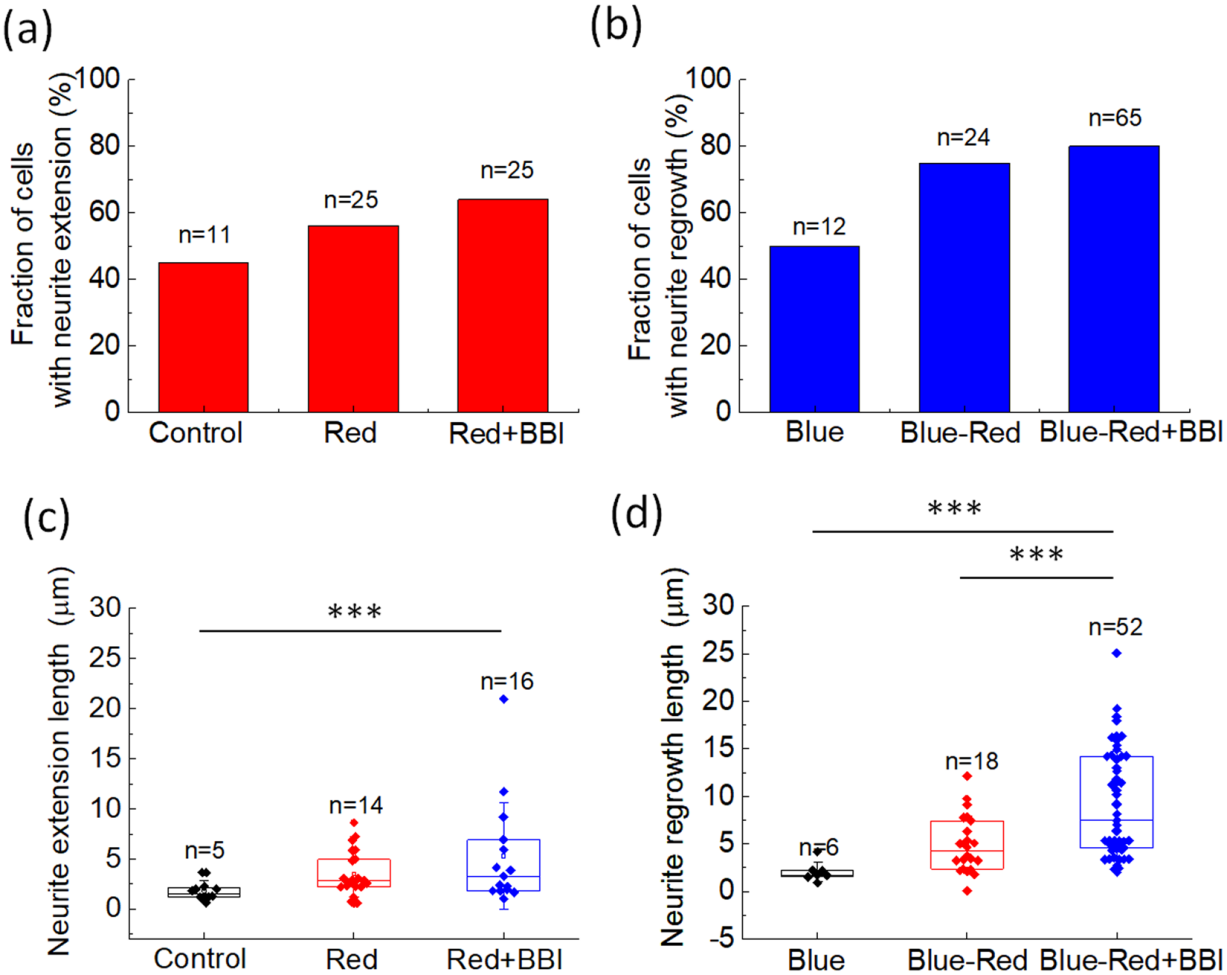
Probability of N2a cells exhibiting (**a**) neurite extension without blue-light stimulation, and (**b**) neurite regrowth after blue light-induced retraction. We also measured neurite extension/regrowth after red-light illumination on the soma for 60 min (**c**) without blue light-induced retraction, and (**d**) after blue light-induced retraction. In these experiments, the concentration of BBI was 50 μM. ***Significant at α < 0.005 (post-hoc Scheffe’s test). The original data for generating (**a,b**) are provided in the Supplementary Information.

We summarize the effects of BBI pre-treatment on red light-induced neurite extension and regrowth in N2a cells in Fig. 4. The extension/regrowth lengths in Fig. 4(c,d) are the same data as those after 60 min of red-light illumination in the temporal plots in Fig. 3(b,c). Figure 4(a,b) show that the red-light illumination increased the probability of regrowth after blue light-induced retraction (from 50% to 75%) more effectively than that of neurite extension (from 45% to 56%). The BBI pre-treatment marginally raised the probability of extension (from 56% to 64%) and regrowth (from 75% to 80%). Nevertheless, the BBI pre-treatment significantly increased the extension and regrowth lengths, as shown in Fig. 4(c,d). The results in Fig. 4 suggest that red-light illumination can trigger neurite regrowth after blue light-induced retraction; and suppressing the activity of myosin II might be helpful for increasing the length of an extending or re-growing neurite.

### Actin activity and calcium influx in neurite regrowth induced by red light

**Figure 5:**
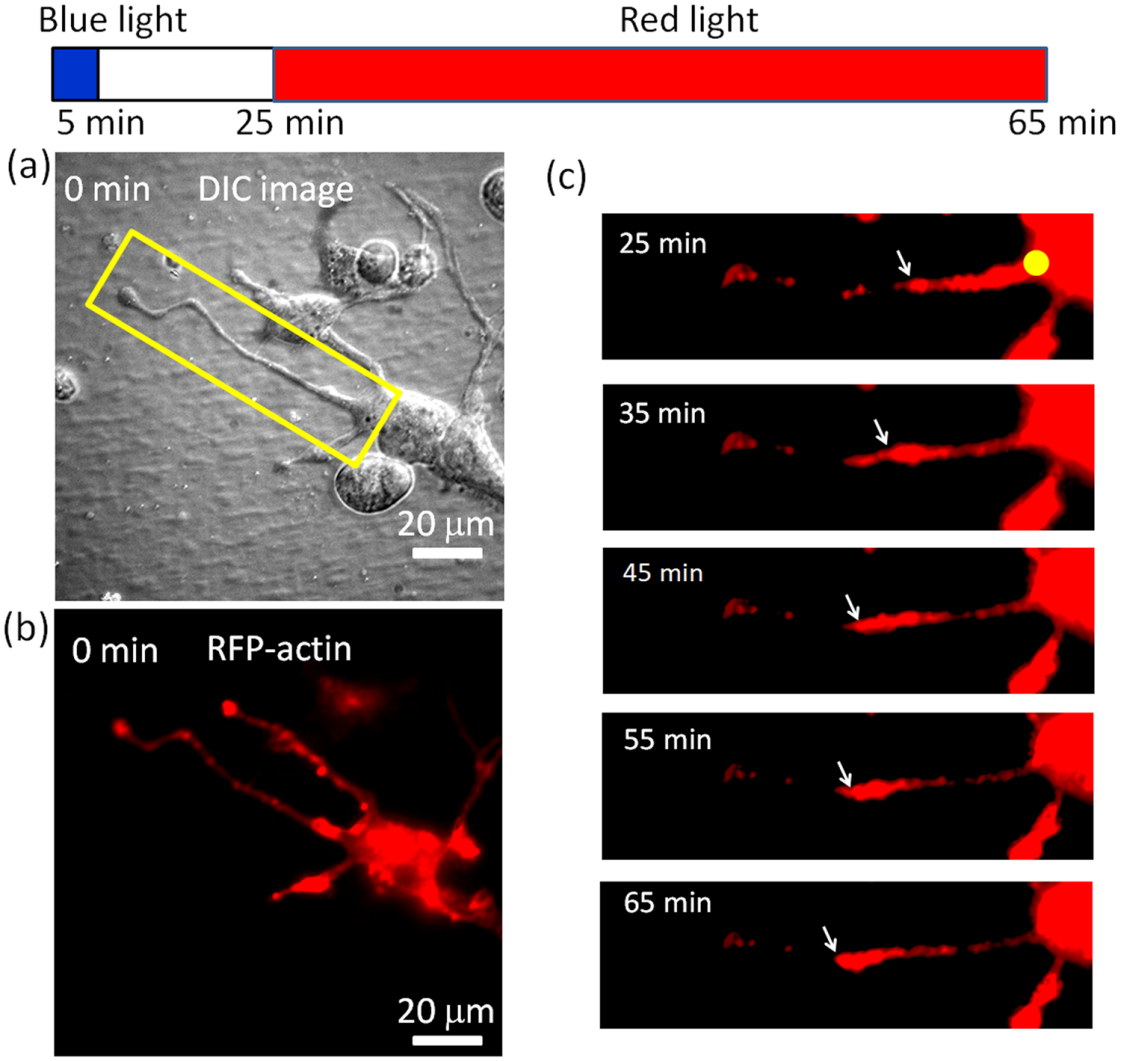
Actin images of a re-growing neurite after the retraction caused by 5 min of blue-light illumination at the tip of an N2a cell. The cell was pre-treated with 50 μM BBI. (**a**) Differential interference contrast image and (**b**) fluorescence image of RFP-tagged actin. (**c**) Time-lapse images of the neurite in the rectangle in (**a**). The arrows indicate the front of an actin aggregation moving in the growing neurite. The yellow dot marks the position of red-light illumination. During the capture of each fluorescence image, the red light was temporarily blocked for ~30 s.

Actin activity plays a crucial role in the growth of neurites^9,11,26^. We used red fluorescent protein (RFP) to mark the actin in neurites of N2a cells in order to observe the actin activity during light-induced neurite regrowth [Fig. 5(a,b)]. The neurite had retracted after 5 min of blue-light illumination on the tip. As shown in Fig. 5(c), we observed an outward movement of actin along with the neurite regrowth. This result indicates that the retraction caused by blue light at the tip did not destroy the cytoskeletal activity in the neurite.

**Figure 6:**
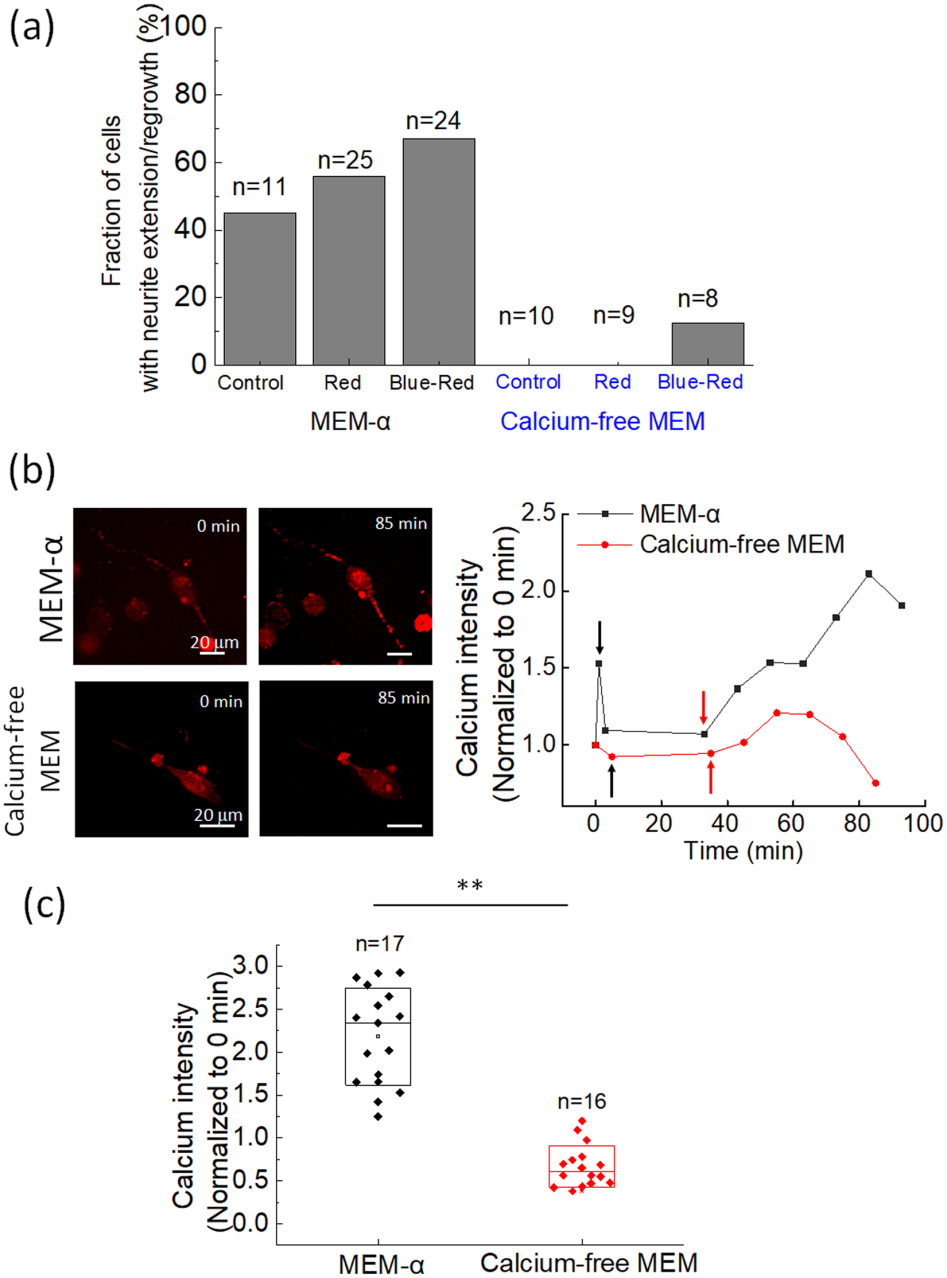
(**a**) Probability of N2a cells with neurite extension or regrowth in MEM-α and calcium-free MEM. ‘Control’ represents cells without any laser light illumination. ‘Red’ represents cells illuminated by red light for 1 h only. ‘Blue-Red’ represents the cells illuminated by blue light for 10 min followed by 60 min of red light. The data for cells in MEM-α were the same as those in Fig. 4(a,b). The original data for generating Fig. (a) are provided in the Supplementary Information. (**b**) Temporal variations of intracellular fluorescence intensity of the calcium ion indicator Rhod-2 AM in N2a cells in MEM-α (black squares) and calcium-free MEM (red dots). The blue-light illumination starts at the beginning. The black arrows mark the end of the blue-light illumination, and the red arrows mark the start of red-light illumination. The red-light illumination was maintained until the end of observation. During the capture of each fluorescence image, the red light was temporarily blocked for ~30 s. (**c**) Intracellular calcium signal after 60 min of red-light illumination for N2a cells in MEM-α and calcium-free MEM. For each cell, the intensity was normalized to its own value at the beginning (0 min). In MEM-α, the average calcium signal was 2.18 times the initial level. In contrast, in calcium-free MEM, the average calcium signal was about 0.67 times the initial level. **P < 0.01 (Wilcoxon–Mann–Whitney test).

Because calcium ions are among the key regulators for neurite growth^4,27^, we suspected that the optically induced neurite regrowth could also be influenced by extracellular calcium ions. We compare the neurite extension and regrowth probability of N2a cells in normal MEM-α culture medium and calcium-free MEM in Fig. 6(a). In the calcium-free MEM, neurite extension without or with red-light stimulation was totally suppressed, and only 12.5% of the tested cells exhibited neurite regrowth after blue light-induced retraction. As shown in Fig. 6(b), in MEM-α, the intracellular calcium ion concentration increased with both blue-light and red-light illumination. After 30 min of red-light illumination, the intracellular calcium ions rose to a level up to twice as high as that prior to illumination. We therefore suspected that photo-induced calcium influx might occur in cells in normal culture medium. In contrast, for the N2a cells cultured in calcium-free MEM, the intracellular calcium concentration increased by less than 20% of the level prior to illumination. We measured the calcium levels after 60 min of red-light illumination of multiple cells and show the results in Fig. 6(c). The average calcium level for cells in MEM-α was increased by 118%. In contrast, the average calcium level for the cells in calcium-free MEM was decreased by 33%. From the results shown in Fig. 6, we presume that calcium influx could be an essential component of optically induced neurite retraction and regrowth.

### Results from primary rat hippocampal neurons

**Figure 7:**
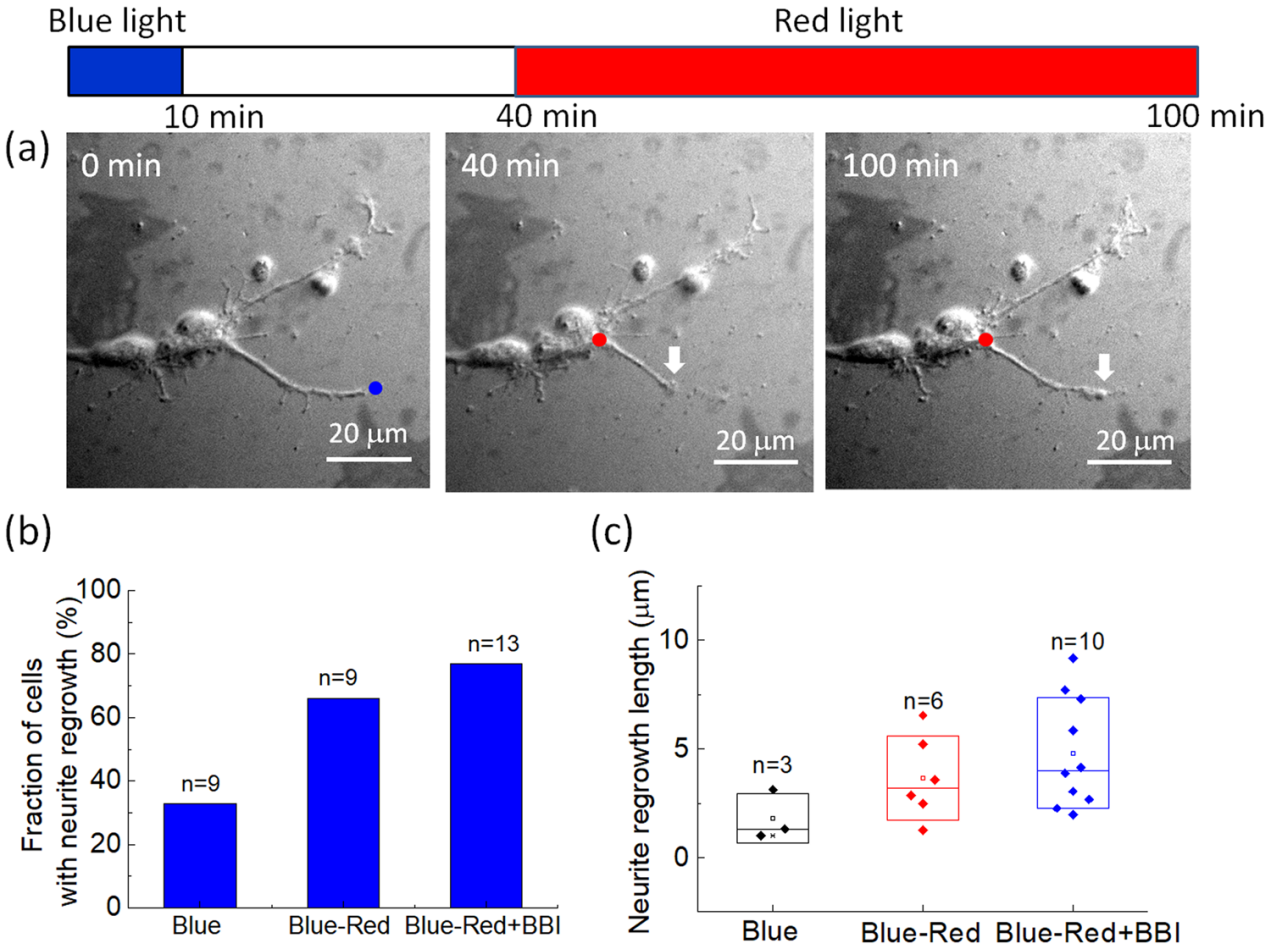
Neurite retraction and regrowth of primary rat hippocampal neuron cells caused by optical stimulation. (**a**) Left, the dot marks the location of blue-light illumination. Middle and right, the dots mark the location of red-light illumination, and the arrow indicates the tip of a neurite. (**b**) Probability of primary rat hippocampal neuron cells with neurite regrowth and (**c**) neurite regrowth lengths for various stimulations after red-light illumination on the soma for 60 min. The original data for generating (**b**) are provided in the Supplementary Information.

In order to verify that neurite regrowth induced by optical stimulation is not limited to N2a cells, we also conducted similar experiments on primary rat hippocampal neuron cells. The results shown in Fig. 7(a) suggest that red-light stimulation of the soma is capable of inducing neurite regrowth after blue light-induced retraction in a rat hippocampal neuron cell. Figure 7(b) shows that red-light illumination increased the probability of regrowth after blue light-induced retraction from 33% to 66%, and BBI pre-treatment further raised the regrowth probability to 77% in rat hippocampal neuron cells. However, there was no significant difference in neurite regrowth lengths among the control and experimental groups Fig. 7(c). The regrowth lengths of the primary neurons were less than those of the N2a cells which were often longer than 10 μm within 1 h of red-light illumination.

**Figure 8:**
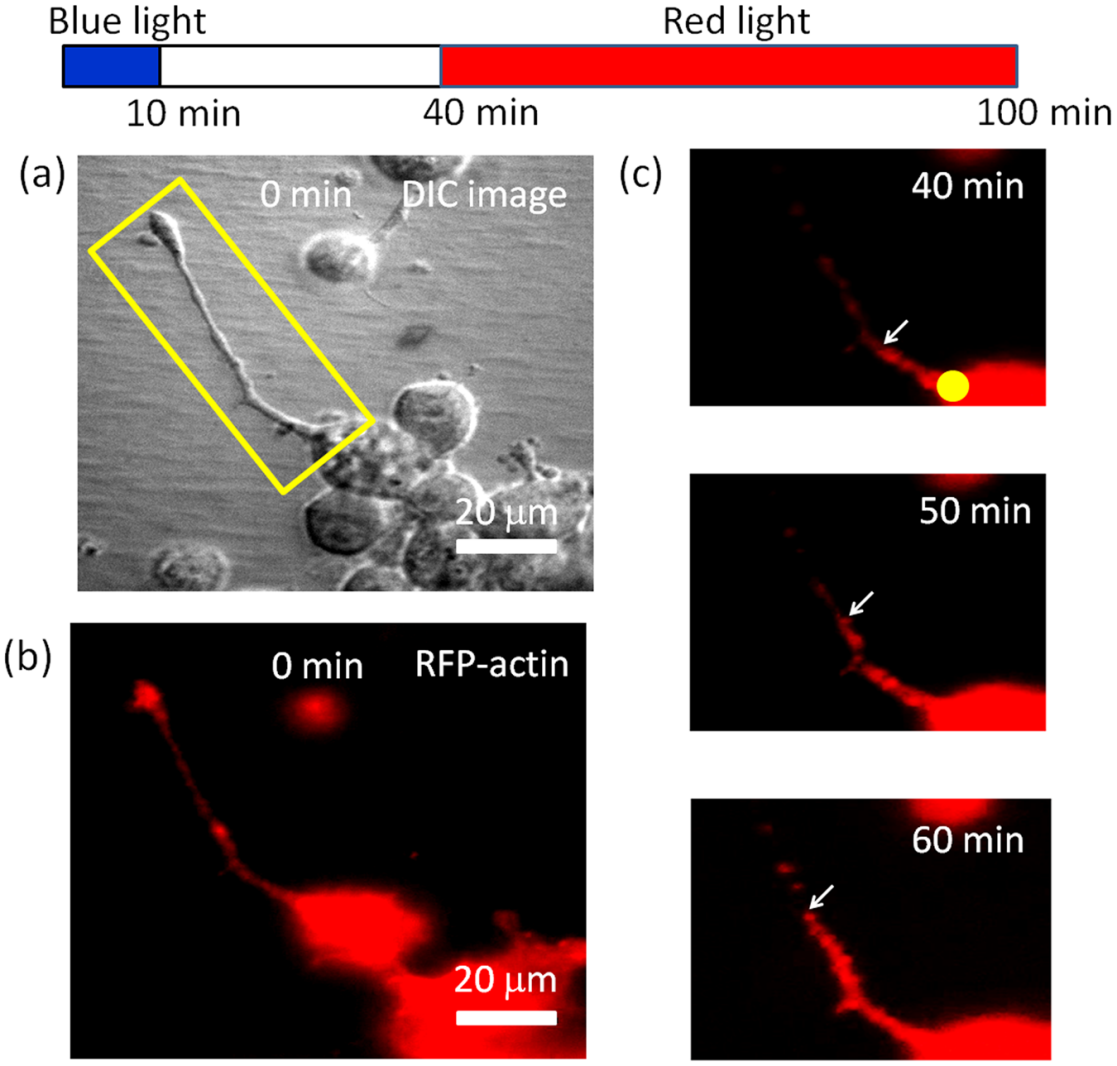
Actin images of a re-growing neurite of a primary rat hippocampal neuron cell after the retraction caused by 10 min of blue-light illumination at the tip. The cell was pre-treated with 50 μM BBI. The yellow dot marks the red-light illumination site. The arrows indicate the front of an actin aggregation. During the capture of each fluorescence image, the red light was temporarily blocked for ~30 s.

We also used RFP-tagged actin to observe actin activity in the re-growing neurites of a rat hippocampal neuron cell [Fig. 8(a,b)]. The time-lapse images in Fig. 8(c) show that actin moved toward the tip of a re-growing neurite as the red-light spot illuminated the soma. This result is similar to what we observed in the N2a cell (Fig. 5).

## Discussion

Inducing the regrowth of damaged neurites is a fundamental prerequisite for repairing degraded or injured neural networks. Electrical stimulation of neurite growth has been proposed and tested for decades^28^, but the requirements of device implantation and a continuous power supply for the implanted device make this technique complicated and expensive in clinical applications. On the other hand, optical stimulation of neurite growth has also been proposed and demonstrated for many years. Neurite guidance is often conducted with a focused near-infrared-light spot or line ahead of the neurite tips or growth cones of axons^12,14,15,20^. Although these works are important for neural cell biology, it can be difficult to locate neurite tips or growth cones in living tissues. The application of unfocused near-infrared light for the repair of peripheral nerves in rabbits^29^ and direct acceleration of neurite growth of mouse dorsal root ganglion neurons by using 645 nm light with a diameter ~7 mm^30^ have been demonstrated. Recently, a review of this field, called photobiomodulation (previously known as low-level light therapy), was provided by Zein *et al*.^31^. The optical intensity used in photobiomodulation studies is typically 5–500 mW/cm^2^. In contrast, because we used focused laser light spots to illuminate the cells, the intensity of red light on cells was 1.27 × 10^5^ mW/cm^2^ while that of blue light was 1.27 × 10^6^ mW/cm^2^.

In the present work, we showed that a focused 650 nm red-light spot on the soma can induce neurite regrowth after retraction caused by 473 nm blue-light illumination at the neurite tip. The neurite retraction could be caused by blue light-induced production of reactive oxygen species (ROS)^22,32^. Because ROS are regarded as important factors in neurodegenerative diseases^33,34^, we considered the blue light-induced neurite retraction pathologically relevant. During retraction, some neurites broke and left residuals on the substrate, such as those seen in the DIC images in Figs. 1(a) and 2(a), and the actin images in Fig. 5(c). On inhibition of myosin II by BBI, we found that the probability of regrowth and the regrowth length of retracted neurites were both increased, implying that the activity of myosin II could be a major source of the retraction force against neurite extension and regrowth. Furthermore, we observed outward movements of actin in the re-growing neurites shown in Figs. 5 and 8 on red-light spot illumination of the soma.

In our experiments, red light-induced neurite extension and regrowth did not occur in calcium-free medium. In normal culture medium, we observed an increase of calcium ion concentration in cells under both blue-light and red-light stimulation. Therefore, we can only conjecture that photo-induced calcium influx is a necessary condition for light-induced neurite activities. At present, we do not have any definitive findings about the initialization mechanisms for this photo-induced calcium influx in neuroblastoma cells.

In conclusion, we have demonstrated that a focused 473 nm blue-light spot illuminating the tip causes neurite retraction, and a focused 650 nm red-light spot illuminating the soma induces neurite regrowth along with outward propagation of actin. The pre-treatment with BBI increases the optically induced neurite regrowth lengths. These optical responses of neurites depend upon a calcium influx.

## Materials and Methods

### Neuronal cells

We used cells from the mouse neuroblastoma N2a cell line (Bioresource Collection and Research Centre, Hsinchu, Taiwan) for the samples. In the present work, we used serum deprivation to differentiate the N2a cells. The cells were placed in a custom-made cell culture chip filled with MEM-α (12571063, Gibco™, ThermoFisher Scientific) with 1% fetal bovine serum (FBS), 1% retinoic acid (Sigma-Aldrich), and 1% antibiotic pen-strep-ampho. The cell-culture chips were kept in a 37 °C incubator filled with 5% CO_2_. After 72 h, the cells developed obvious neurites. Then we replaced the medium in the chip with MEM-α with 10% FBS and 1% antibiotic pen-strep-ampho for the later experiments. In the experiment for testing the role of extracellular calcium ions in optically induced neurite regrowth, the medium in the chip of differentiated N2a cells was calcium-free MEM (11380037, Gibco™, ThermoFisher Scientific). For light stimulation experiments, we placed the cell culture chip on the sample stage of an upright microscope (Eclipse LV150, Nikon, Tokyo, Japan) within a 37 °C temperature-controlled cage. The cells could be kept alive for more than 8 h on the microscope stage. All the experiments were conducted within 6 hours in the MEM-α or the calcium-free MEM. In other words, in each culture chip we observed 2–3 cells only, and then we used the cells in another culture chip. The control and treatment experiments were conducted alternatively without specific selection of the time after differentiation. We used one batch of differentiated N2a cells within three days, and then used a newly differentiated batch of cells.

We also used primary rat neuron cells as a sample. The hippocampal neurons were prepared from rat embryos on E17 as previously described^35^; and were cultured in neurobasal medium supplemented with Gem21 NeuroPlex (GEMINI Bio-Products) for 24 h in the cell culture chip. The cell-culture chips were kept in a 37 °C incubator filled with 5% CO_2_. For light stimulation experiments, we placed the cell culture chip on the sample stage of an upright microscope as stated in the previous paragraph. We used Hank’s Balanced Salt Solution (14025092, Gibco™, ThermoFisher Scientific) as the medium in the light stimulation experiments of the primary rat neuron cells, and conducted the experiments within 6 hours. The control and treatment experiments were conducted alternatively without specific selection of the time after acquiring the primary cells. One batch of the primary rat neuron cells were used within one day.

### Laser light stimulation

We coupled two continuous-wave laser beams of wavelengths 473 nm (ALBB-030–5010, Onset, New Taipei City, Taiwan) and 650 nm (AM-BQ-200, Onset) into the epi-illumination port of an upright optical microscope (Eclipse LV150, Nikon). The microscope was enclosed in a temperature-controlled cage and kept at 37 °C. The laser beams were focused onto the cells with a 40×, numerical aperture 0.8 objective. The diameter of the focused spots of 473 nm and 650 nm light was 4.0 μm and 5.0 μm, respectively, measured on the same image plane on which we observed the cells. When measuring the spot diameters, the laser power was attenuated such that the image sensor was not saturated. For the experimental results reported in the present work, the 473 nm light power on the cell was 160 μW; and that of the 650 nm light was 25 μW unless otherwise indicated. The laser power was measured after the objective by using a handheld power meter (LaserCheck, Coherent, Santa Clara, CA, USA).

In order to compare the effects of various laser wavelengths (550, 600, and 700 nm), we employed a supercontinuum laser as the light source (SC-450-2, Fianium, Southampton, UK). The specific wavelengths were selected by using interference filters with a 10 nm bandwidth. The diameter of the focused spots of the 550, 600, and 700 nm light was 4.2, 5.0, and 5.0 μm respectively.

### Motor protein inhibitors

We used the following inhibitors of motor proteins individually to treat the N2a cells. The myosin II inhibitor blebbistatin (BBI; Sigma-Aldrich, St. Louis, MO, USA) was prepared in the culture medium at a concentration of 50 μM, following some previous works discussing the roles of myosin II in neurite growth^36,37^. We dissolved BBI in DMSO at a concentration of 10 mM, and then diluted it into the culture medium at a volume ratio of 1: 200. The cells were pre-treated with BBI for 30 min before optical stimulation. Although blue light can inactivate BBI^38^, in the present work the blue light was focused at the tip of a neurite with a diameter ~4.0 μm and the intensity was much lower than the effective intensities reported in^38^. Therefore, we do not think that the blue light inhibited BBI inside cells. The myosin light chain kinase inhibitor ML7 (abcam, San Francisco, CA, USA) was dissolved in DMSO (100 mM) and then diluted into the culture medium at a concentration of 10 μM^39^. The kinesin-5 inhibitor monastrol (Sigma-Aldrich) was dissolved in DMSO (10 mM) and then diluted into the culture medium at a concentration of 100 μM^40^. The cytoplasmic dynein inhibitor ciliobrevin D (Sigma-Aldrich) was dissolved in DMSO (100 mM) and then diluted into the culture medium at a concentration of 200 μM^41^. Following the parameters used in the literature, the N2a cells were pre-treated with ML7, monastrol, and ciliobrevin D for 1, 4, and 1 h, respectively, before optical stimulation. The highest DMSO concentration in the medium was 1% (v/v) in the monastrol experiment. Therefore, we also observed the blue light-induced neurite retraction in the medium containing 1% (v/v) DMSO. The light stimulation experiments were conducted with one inhibitor at a time without specific selection of the time after differentiation.

### Fluorescence imaging

For the observation of intracellular actin in live cells, we utilized PolyFect Transfection Reagent (QIAGEN, Hilden, Germany) to transfect the cells with the pTagRFP-actin vector (Evrogen, Moscow, Russia). The exposure time for an actin image was 1.0 s. For the observation of intracellular calcium ions in live cells, we used the calcium ion indicator Rhod-2 AM (Cayman Chemical, Ann Arbor, MI, USA) to stain the cells for 1 h, and then replaced the medium. The exposure time for a calcium image was 1.0 s. We set the electron-multiplying gain of the camera as 10 for acquiring the calcium images. During the capture of each actin or calcium image, the red light was temporarily blocked for ~30 s. The average pixel value (after background subtraction) within a cell normalized to that of the same cell measured before any optical stimulation was recorded as the relative intracellular calcium level.

The fluorescence images were acquired using an epifluorescence microscope (Eclipse Ti-E, Nikon, Tokyo, Japan). The images were captured by a 14-bit electron-multiplying charge-coupled device (EMCCD) camera (DU-885, Andor, Belfast, Northern Ireland) cooled down to −60 °C. The fluorescence intensity of individual cells was quantified using ImageJ (http://rsb.info.nih.gov/ij/).

### Data analysis

Because of the long observation time, typically we could only measure the neurite extension/regrowth lengths of 2–3 cells in one experiment. Therefore, we performed nearly *n*/2 independent experiments for each experimental group. All the data are presented as mean ± standard deviation. The comparisons among data of more than two groups were conducted with one-way analysis of variance (ANOVA). A *P*-value less than 0.05 was considered significant. Then the differences between two specific groups were checked with post-hoc Scheffe’s test. An α-value less than 0.05 was considered as a significant difference in the results of Scheffe’s test. The comparison of calcium intensity in Fig. 6(c) was conducted with the Wilcoxon–Mann–Whitney test.

## Supplementary information

Supplementary Information
