# Supplementary material for: Neurite regrowth stimulation by a red-light spot focused on the neuronal cell soma following blue light-induced retraction
Source: Sci Rep. 2019 Dec 3;9:18210. doi: 10.1038/s41598-019-54687-w (PMC6890775; doi:10.1038/s41598-019-54687-w)
Supplement: Supplementary file 1 — Supplementary Information [file 41598_2019_54687_MOESM1_ESM.pdf]

Supplementary Information

Neurite regrowth stimulation by a red-light spot focused on the neuronal cell soma following blue light-induced retraction

Yu-Chiu Kao,[1] Yu-Cing Liao,[2] Pei-Lin Cheng,[3] and Chau-Hwang Lee[1, 2*]

[1]Research Center for Applied Sciences, Academia Sinica, Taipei 11529, Taiwan

[2]Institute of Biophotonics, National Yang-Ming University, Taipei 11221, Taiwan

[3]Institute of Molecular Biology, Academia Sinica, Taipei 11529, Taiwan

***Correspondence:** Chau-Hwang Lee, Research Center for Applied Sciences, Academia Sinica, 128 Sec. 2, Academia Rd., Taipei 11529, Taiwan. E-mail: clee@gate.sinica.edu.tw

	Retraction ($-\Delta L > 5$ μm after 30 min)	No retraction	Cell blebbing or swelling
Control	3	23	0
Blue light only	36	1	2
DMSO	27	4	1
Blebbistatin	16	44	13
ML7	9	28	6
Monastrol	12	6	2
Ciliobrevin D	15	7	1

Supplementary Table 1. Numbers of N2a cells exhibiting neurite retraction, no retraction or cell blebbing or swelling caused by blue light with the pre-treatments of blebbistatin, ML7, monastrol, and ciliobrevin D. A retraction event was only recognized for neurites which retracted more than 5.0 μm after 10 min of blue-light illumination plus a 30-min duration without illumination. The probabilities of cells with neurite retraction are shown in Fig. 1(b).

Wavelength (nm)	Regrowth (ΔL > 1 μm)	No regrowth	Cell blebbing or swelling
Control	6	5	1
550	18	11	0
600	9	4	0
650	18	4	2
700	16	12	0

Supplementary Table 2. Numbers of N2a cells exhibiting neurite regrowth, no regrowth or cell blebbing or swelling under various wavelengths of illumination on soma. A regrowth event was only recognized for neurites with growth lengths of more than 1.0 μm after 60 min of illumination. The probabilities of cells with neurite regrowth are shown in Fig. 2(b).

Power	Regrowth (ΔL > 1 μm)	No regrowth	Cell blebbing or swelling
Control	6	5	1
25 μW	18	4	2
90 μW	14	4	1
1.5 mW	8	2	2

Supplementary Table 3. Numbers of N2a cells exhibiting neurite regrowth, no regrowth or cell blebbing or swelling under various power levels of 650 nm light. A regrowth event was only recognized for neurites with growth lengths of more than 1.0 μm after 60 min of illumination. The probabilities of cells with neurite regrowth are shown in Fig. 2(d).

	Extension ($\Delta L > 1\ \mu m$)	No extension	Cell blebbing or swelling
Control	5	5	1
Red	14	11	0
Red+BBI	9	1	0

Supplementary Table 4. Numbers of N2a cells exhibiting neurite extension, no extension or cell blebbing or swelling without the blue-light stimulation. An extension event was only recognized for neurites with growth lengths of more than 1.0 μm after 60 min of illumination. The probabilities of cells with neurite extension are shown in Fig. 4(a).

	Regrowth ($\Delta L > 1\ \mu m$)	No regrowth	Cell blebbing or swelling
Blue	6	5	1
Blue-Red	18	4	2
Blue-Red +BBI	52	11	0

Supplementary Table 5. Numbers of N2a cells exhibiting neurite regrowth, no regrowth or cell blebbing or swelling after the blue-light-caused retraction. A regrowth event was only recognized for neurites with growth lengths of more than 1.0 μm after 60 min of illumination. The probabilities of cells with neurite regrowth are shown in Fig. 4(b).

	Extension or regrowth ($\Delta L > 1\ \mu m$)	No extension nor regrowth	Cell blebbing or swelling
**Control (MEM-α)**	5	5	1
**Red (MEM-α)**	14	11	0
**Blue-Red (MEM-α)**	18	4	2
**Control (Ca free MEM)**	0	10	0
**Red (Ca free MEM)**	0	9	0
**Blue-Red (Ca free MEM)**	1	6	1

Supplementary Table 6. Numbers of N2a cells with neurite extension or regrowth, no extension nor regrowth or cell blebbing or swelling in MEM-α and calcium-free MEM. "Control" represents the cells without any laser-light illumination. A regrowth or extension event was only recognized for neurites with growth lengths of more than 1.0 μm after 60 min of illumination. The probabilities of cells with neurite extension or regrowth are shown in Fig. 6(a).

	Regrowth ($\Delta L > 1\ \mu m$)	No regrowth	Cell blebbing or swelling
Blue	3	4	2
Blue-Red	6	2	1
Blue-Red +BBI	10	2	1

Supplementary Table 7. Numbers of rat hippocampal neuron cells with neurite regrowth, no regrowth or cell blebbing or swelling. A regrowth event was only recognized for neurites with growth lengths of more than 1.0 μm after 60 min of illumination. The probabilities of cells with neurite regrowth are shown in Fig. 7(b).
